# Landscape of Infiltrated Immune Cell Characterization in Uveal Melanoma to Improve Immune Checkpoint Blockade Therapy

**DOI:** 10.3389/fimmu.2022.848455

**Published:** 2022-03-02

**Authors:** Xiaohui Lv, Min Ding, Yan Liu

**Affiliations:** Department of Ophthalmology, Affiliated Hospital of Weifang Medical University, Weifang, China

**Keywords:** infiltrated immune cells, tumor microenvironment, prognosis, immunotherapy, uveal melanoma

## Abstract

**Background:**

Numerous studies indicated that tumor-infiltrated immune cells (TIC) in the microenvironment are substantially linked to immunotherapy response and cancer prognosis. However, systematic studies of infiltrated immune cell characterization in uveal melanoma (UM) for prognosis and immune checkpoint blockade therapy are lacking.

**Methods:**

UM datasets were extracted from open access resources (TCGA and GEO databases). The tumor-infiltrated immune cells in the microenvironment were decoded by using the CIBERSORT algorithm, which was further applied to classify UM patients into subgroups using an unsupervised clustering method. The Boruta algorithm and principal component analysis were used to calculate the TIC scores for UM patients. Kaplan–Meier curves were plotted to prove the prognostic value of TIC scores. Besides, the correlations of the TIC score with clinical features, mutated characteristics, and the immune therapeutic response were subsequently investigated.

**Results:**

As a result, we defined three subtypes among 171 UM patients according to the TIC profiles and then calculated the TIC score to characterize the immune patterns for all patients. We discovered that high-TIC score patients with low BAP1 and high EIF1AX mutations have a better prognosis than low-TIC score patients. Activation of immune inflammatory response and increase in immune checkpoint-related genes in high-TIC score patients may account for good prognosis and immunotherapy response. Three melanoma cohorts received immunotherapy, proving that high-TIC score patients have substantial clinical and immune therapeutic improvements. Besides, several potential therapeutic agents were identified in the low-TIC score group.

**Conclusion:**

Our study afforded a comprehensive view of infiltrated immune cell characterization to elucidate different immune patterns of UM. We also established a robust TIC-score signature, which may work as a prognostic biomarker and immune therapeutic predictor.

## Background

Uveal melanoma (UM) is a highly fatal intraocular tumor that develops from the transformation of the eye’s malignant melanocytes. The choroid tissue is the most common site for UM initiation, with the iris (3%–5%) and ciliary body (5%–8%) accounting for the remaining occurrences (1, 2). Despite the incidence of UM being rare, about 0.06%–0.07‰ incidence was reported in the United States; approximately half of patients will die from systemic metastases (3). Because UM often micro-metastasizes soon after its initial diagnosis, traditional treatments such as enucleation, resection, and radiation therapy are constantly disappointed (4, 5). Yet, there are no systemic therapies that have been proven to effectively improve the clinical outcomes of metastatic UM. Although immune checkpoint inhibitors like anti-PD1, anti-PDL1, and anti-CTLA4 have been effectively employed in metastatic cutaneous melanoma, one of the biggest drawbacks for application of immunotherapy in UM is that only approximately 0% to 6% of response rate was observed in clinical use (6–8). The negative reaction to immune checkpoint blockade in UM reveals a gap in our understanding of how advanced UM evades immune surveillance or generates tolerance. Therefore, exploration of immune cell characterization and identification of novel biomarkers for immunosuppression of UM are urgently required.

The tumor microenvironment (TME) includes abundant non-tumor cells such as immune cells, adipocytes, fibroblasts, endothelial cells, and mesenchymal cells that surrounded the tumor cells and is recognized to have an important role in tumor development and immunological heterogeneity (9–11). The immune components in TME like infiltrated immune cells and cytokines have a significant effect in prognosis, drug resistance, and immunotherapy response of various tumors (12–14). Generally, the high level of infiltrated immune cells of TME in cancers is associated with good prognosis, whereas the infiltration of immune cells in UM indicates a poor outcome (15, 16). For example, Narasimhaiah et al. recently observed that the increased infiltration of T lymphocytes and macrophages is linked to the progression of metastatic UM and associated with a poor prognosis (17). It is universally accepted that the eye is an immune-privileged organ where numerous immunosuppressive elements exist in the microenvironment. This immunosuppressive environment can hinder the traffic of activated T cells into the tumor tissues, exhaust the existing effective T cells, and eventually alter the phenotype of infiltrated T cells (18, 19). Recent single-cell analysis of UM demonstrated that the main infiltrated T lymphocytes in TME are CD8+ T cells, and an exhausted subtype accounts for a great proportion of CD8+ T cell subsets (7, 20). Therefore, in order to spark new ideas for therapy and prognosis of UM, we need to comprehensively recognize the characterization of infiltrated immune cells and deeply understand the microenvironment.

Due to the advances in next-generation sequencing (NGS) technology, large-scale transcriptome profiles are generated and deposited in open repositories, such as TCGA and GEO databases, which have revealed a wealth of biological knowledge on tumorigenesis of cancers. For example, Wang and colleagues established an immune-associated prognostic score based on 22 breast cancer cohorts, which could be used to predict overall survival and response to immunotherapy (21). Yang et al. discovered that highly expressed immune-related genes were linked to a favorable prognosis in high-grade carcinomas (22). Besides, Newman et al. firstly developed CIBERSORT in 2015, a novel deconvolution methodology that uses transcriptome profiles to decode immune cell infiltration (ICI) profiles (23). Finally, we can accessibly classify UM patients by using ICI profiles and contributing to the personalized treatment as well as increasing the average benefit accordingly.

Therefore, we performed CIBERSORT to systematically examine the characteristics of 22 kinds of tumor-infiltrated immune cells in the UM microenvironment based on the bulk RNA sequencing datasets. Combined with an unsupervised clustering method, UM patients were successfully categorized into distinct subgroups with varied clinical features and survival events based on ICI profiles. To summarize, we eventually developed the TIC scores to define the diverse immunological landscapes in this study, which may accurately predict UM patients’ prognosis and immunotherapy response.

## Materials and Methods

### UM Data Acquiring

**Figure 1:**
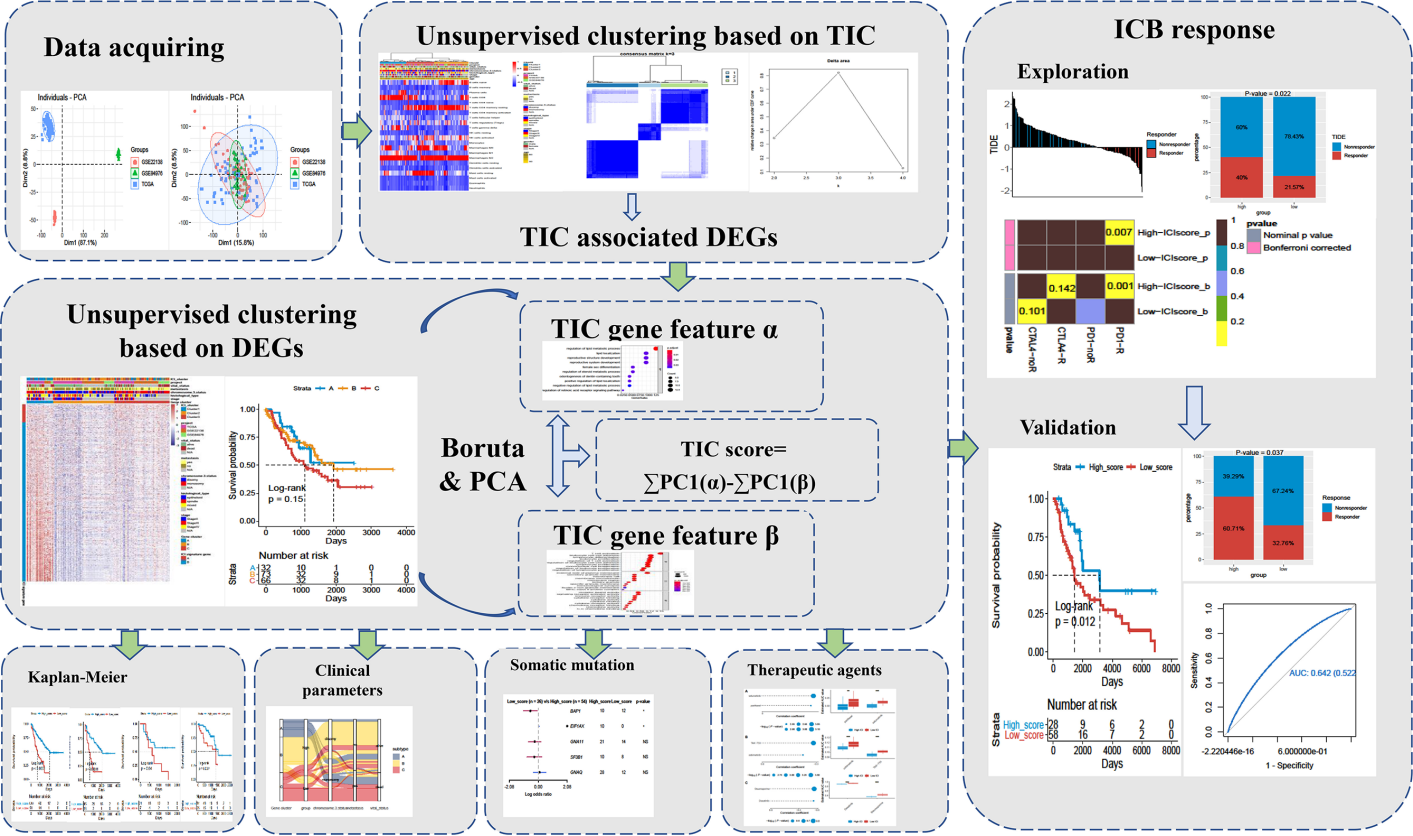
The complete research workflow.

The bulk RNA-seq of uveal melanoma (UM) data as well as clinical information were extracted from open access resources (TCGA and GEO database). The uveal melanoma dataset (TCGA-UVM) was acquired from UCSC Xena (http://xena.ucsc.edu). The UM-associated transcriptomes (GSE84976 and GSE22138) were obtained from the GEO website (https://www.ncbi.nlm.nih.gov/geo). Then, fragments per kilobase per million (FPKM) format of gene expression in TCGA-UVM was converted into transcripts per kilobase million (TPMs). The raw count of GEO datasets was also transformed to the TPMs format. Next, these datasets were merged into a large metadata by the “ComBat” algorithm, which can decrease batch effects caused by different platforms and non-biological technical biases. A simplified workflow for the current investigation is depicted in **Figure 1**.

### Tumor-Infiltrated Immune Cell Estimation and Clustering

Based on the 1,000 permutations of LM22 signature, the “CIBERSORT” method was used to calculate the percentage of 22 different types of immune cells in the UM TME. Next, the “ConsensusClusterPlus” program was applied to stratify the UM patients into three separate subgroups in order to show the biological importance of different immune cell infiltrations. This program employed the unsupervised clustering “Pam” approach and Euclidean and Ward’s linkage, which was performed 1,000 times to confirm classification consistency.

### Differently Expressed Gene-Associated Clusters

UM patients in metadata were categorized into subgroups based on infiltrated immune cells. We used the “limma” method to screen immune cell-related DEGs and set the significant cutoff criteria to *p* < 0.05 (Adjust) and |log2FC| ≥1.5. To further investigate the underlying biological mechanism of DEGs, GO and KEGG annotation were conducted and the enriched items with adjust *p* < 0.05 were considered as significant.

### Tumor-Infiltrated Immune Cell Score Estimation

To begin with, the expressed values of differently expressed genes were used to divide the UM patients by using unsupervised clustering. Genes with positive and negative correlations to genomic clusters were respectively defined as TIC gene features α and β. Next, the important gene sets in features α and β were identified by using the Boruta algorithm, which subsequently employed PCA calculation. Finally, principal component 1 was respectively extracted to represent the feature score. TIC scores in UM samples were calculated by the following formula: Σ*PC*1 (α) - Σ*PC*1 (β).

### Somatic Mutation Profile Analysis

UM patients in the TCGA-UVM cohort had their mutation data deposited in the UCSC Xena website. We downloaded the Mutation Annotation Format file and analyzed it using the “Maftools” package. OncoPrint plots were used to display the mutation landscape of high- and low-TIC score subgroups. The different alteration frequencies between high- and low-TIC score subgroups were explored. Besides, Maftools was used to compute tumor mutational burden (TMB) in the TCGA-UVM cohort; the relationship between TMB and TIC score was then investigated.

### Immune Checkpoint Therapy Datasets

To test the prognostic value and immune checkpoint therapy response of TIC scores, three independent melanoma cohorts, namely, TCGA-SKCM, GSE35640, and GSE78220, were downloaded and analyzed. The transcriptional profile of TCGA-SKCM and related information were acquired from the UCSC Xena website. The gene expression profiles of GSE35640 and GSE78220 as well as related information were obtained from the GEO database. In all, a number of 169 melanoma patients who accepted immune checkpoint therapy were analyzed to measure the TIC score.

### Potential Therapeutic Agent Prediction

To determine the possible therapeutic agents, three different drug response databases, namely, CTRP (https://portals.broadinstitute.org/ctrp.v2.1), PRISM (https://www.theprismlab.org/), and GDSC (https://www.cancerrxgene.org/), were used to investigate the associations between drug response and TIC scores. Firstly, Spearman correlation between TIC scores and AUC values was used to identify potential therapeutic agents (CTRP: *r* < −0.20; PRISM: *r* < −0.20; GDSC: *r* < −0.40). Next, differential drug response between the high-TIC score group (upper decile) and the low-TIC score group (lower decile) was determined to identify compounds with a higher AUC in the low-TIC score group.

### Statistical Analysis

All statistical tests were conducted using R software version 3.6.0 with packages. The “ConsensusClusterPlus” package was used to conduct clustering. The “Boruta” package was performed to Boruta algorithm. The “CIBERSORT” package was used to estimate the immune cells. The “Survival” package made it possible to perform Kaplan–Meier (KM) survival analysis. The correlation analyses were assessed by Spearman test. Subgroup analyses was conducted by Kruskal−Wallis or Wilcoxon test. The chi-square test was used to analyze the different mutations across groups. *p* < 0.05 or Adjust *p* < 0.05 was considered to be statistically significant.

## Results

### Landscape of the Infiltrated Immune Cells in UM

**Figure 2:**
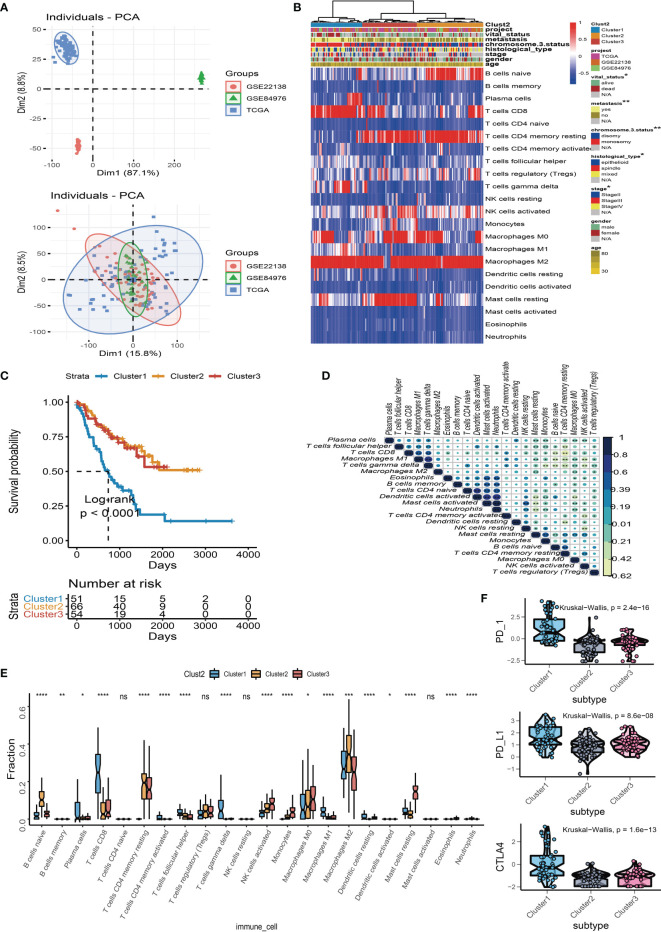
Landscape of the infiltrated immune cell characterization in uveal melanoma (UM). **(A)** The distribution of principal component analysis for UM (TCGA-UVM, GSE84976, and GSE22138) before and after removal of batch effect. **(B)** Clustering of tumor-infiltrated immune cells in 171 UM patients using an unsupervised clustering method. Twenty-two kinds of infiltrated immune cells are shown by rows, and UM samples are represented by columns. **(C)** Kaplan–Meier plots for overall survival time of three subtypes of UM patients. **(D)** The correlation analysis of 22 kinds of infiltrated immune cells. **(E)** Box plots of infiltrated immune cells in three subtypes. **(F)** Box plots of immune checkpoint genes (PD-1, PD-L1, and CTLA-4) in three subtypes. *p < 0.05; **p < 0.01; ***p < 0.001; and ****p < 0.0001. NS means “none significance”.

A total of 171 uveal melanoma (UM) samples were pooled into a big cohort that was studied in this research. We firstly removed the batch effect caused by the various platforms *via* the “ComBat” method. Before the removal of batch effect, the clusters of different platforms were clustered more closer than after removal (**Figure 2A**). Next, the CIBERSORT method was performed to determine the percentage of 22 immune cells in the immune microenvironment of UM. The UM patients were further categorized into subgroups based on the similar proportions of infiltrated immune cells. A steadily increasing trend in the cumulative distribution function (CDF) value was seen as a sign of stable clustering. We finally discovered three stable subtypes by applying an unsupervised clustering (*k* = 3), which contained Cluster 1 (51 UMs), Cluster 2 (66 UMs), and Cluster 3 (54 UMs). The relationship between subtypes and clinical characteristics was investigated and displayed in a complete heatmap (**Figure 2B**). The chi-square test showed significant variations in vital status, metastasis, chromosome 3 status, stage, and histological type among subtypes. Moreover, KM curves revealed that UM patients in Cluster 1 have a worst overall survival rate among subtypes with log rank test *p* < 0.0001 (**Figure 2C**). We further analyzed the immune cell composition of the UM microenvironment to better understand the fundamental biological distinctions that led to different clinical presentations. The association heatmap visualized the comprehensive interaction of immune cells in the UM microenvironment (**Figure 2D**). Among these subtypes, Cluster 1 had a high number of plasma cells, CD8 T cells, activated CD4 T cells, follicular helper T cells, gamma delta T cells, M1 macrophages, and resting dendritic cells, while Cluster 2 and Cluster 3 had a high number of naïve B cells, memory B cells, resting CD4 memory T cells, activated NK cells, monocytes, M0/M2 macrophages, activated dendritic cells, resting mast cells, eosinophils, and neutrophils (**Figure 2E**). In addition, the expressions of three essential immune checkpoint molecules (PD-1, PD-L1, and CTLA-4) were investigated in each subgroup. The Kruskal–Wallis test indicated that Cluster 1 had a higher expression of PD-1, PD-L1, and CTLA-4 than Cluster 2 and Cluster 3 (**Figure 2F**).

### Immune Gene-Related Cluster

**Figure 3:**
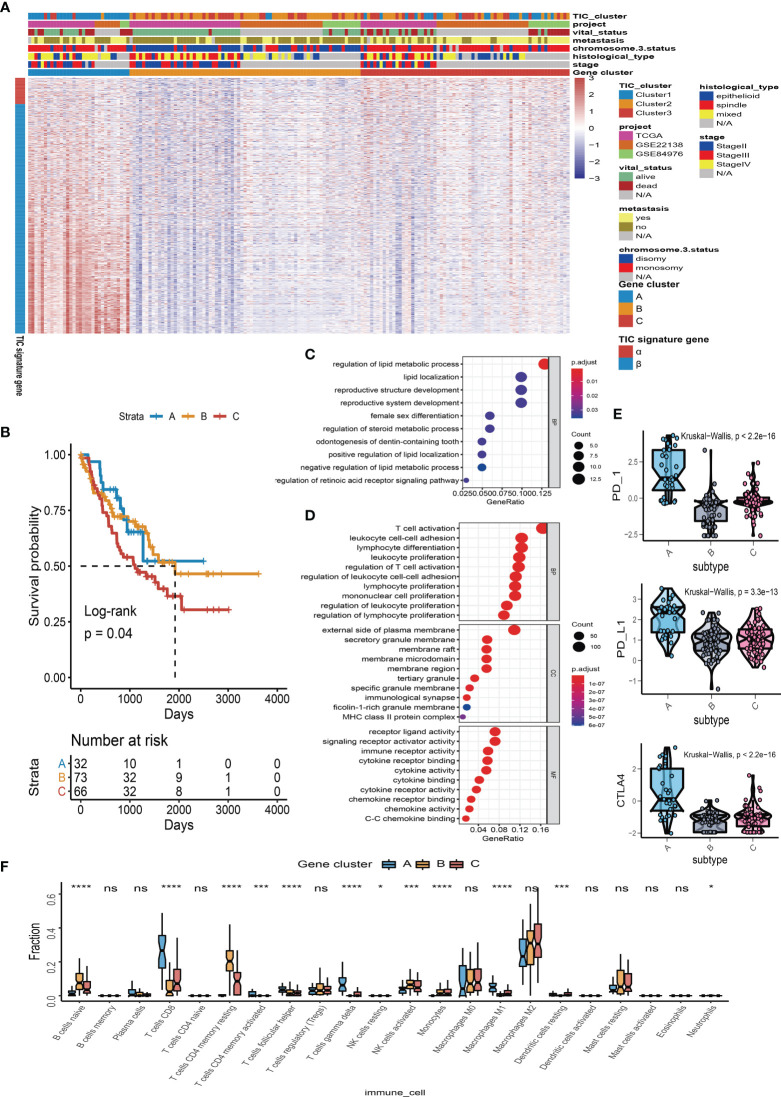
Immune gene-related cluster. **(A)** UM patients were classified into three subgroups (Clusters A to C) by using unsupervised clustering of DEGs among three TIC subtypes. Clustering analysis of prognostic ferroptosis-related genes (FRGs) in melanoma. **(B)** Kaplan–Meier plot for the three subgroups of patients. **(C)** Gene Ontology (GO) enrichment of feature α gene set. **(D)** GO enrichment of feature β gene set. **(E)** Box plots of immune checkpoint genes (PD-1, PD-L1, and CTLA-4) in three gene-related clusters. **(F)** Box plots of infiltrated immune cells in three gene-related clusters. *p < 0.05; ***p < 0.001; and ****p < 0.0001. NS means “none significance”.

We used “limma” differential analysis to evaluate the DEGs among these subgroups to uncover the underlying biological properties of diverse immune phenotypes. A total of 1,054 DEGs were identified for unsupervised clustering analysis. UM samples were subsequently divided into three immune gene-related clusters (A–C). Cluster B was linked to a better prognosis with a log rank test *p* = 0.04 (**Figure 3B**). The feature α gene set was made up of 108 DEGs that were positively associated with the gene cluster, whereas feature β was made up of 946 DEGs that were negatively associated with the gene cluster. The relationship between the immune gene-related cluster and clinical characteristics was depicted in a heatmap of DEG expression (**Figure 3A**). The significantly enriched GO terms in feature α and feature β gene sets are illustrated in **Figures 3C, D**. Besides, the expression levels of PD-1, PD-L1, and CTLA-4 were investigated in each cluster. The Kruskal–Wallis test indicated that the expressions of PD-1, PD-L1, and CTLA-4 were generally higher in Cluster A than those in Cluster B and Cluster C (**Figure 3E**). Among the three gene clusters, Cluster A had a high infiltration of CD8 T cells, follicular helper T cells, gamma delta T cells, and M1 macrophages, whereas Cluster B and Cluster C had a high number of naïve B cells, resting memory CD4 T cells, activated NK cells, monocytes, resting dendritic cells, and neutrophils (**Figure 3F**).

### TIC Score Estimation

**Figure 4:**
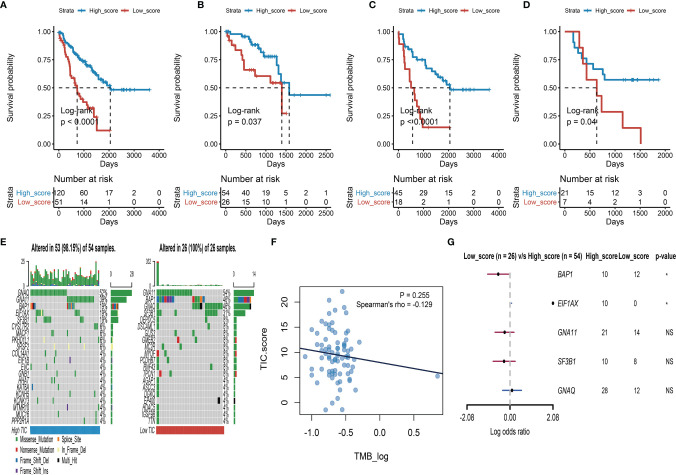
Survival analysis and somatic mutation. **(A)** Kaplan–Meier (KM) plot of high-TIC score and low-TIC score groups in all UM patients. **(B)** KM plot of high-TIC score and low-TIC score patients in the TCGA-UVM cohort. **(C)** KM plot of high-TIC score and low-TIC score patients in the GSE22138 cohort. **(D)** KM plot of high-TIC score and low-TIC score patients in the GSE84976 cohort. **(E)** The top 20 mutant genes in the high-TIC score and low-TIC score groups. **(F)** The correlation analysis between tumor burden mutation (TMB) and TIC scores. **(G)** Different somatic mutations between high-TIC score and low-TIC score groups.

To find the qualified gene indicator for the computation of TIC scores, we used the Boruta method to respectively select the important gene sets in feature α and feature β. A total of 109 DEGs (**Table S1**) consisting of 13 DEGs in feature α and 96 DEGs in feature β were selected for PCA calculation. Furthermore, we acquired the TIC scores for each UM patient based on the earlier formula. After that, the optimal cutoff value of TIC score was used to divide UM patients into two subgroups: high score and low score. Log-rank test in KM curve suggested that high-TIC score patients had a good overall survival time than those in the low-TIC score group (**Figure 4A**). To assess the robustness of TIC scores, stratification analyses were also conducted. TCGA-UVM, GSE22138, and GSE84976 were accordingly separated into high-score and low-score groups. KM curves prove that high-TIC score patients had a good prognosis than low-TIC score patients regardless of TCGA-UVM (**Figure 4B**), GSE22138 (**Figure 4C**) and GSE84976 (**Figure 4D**).

### Somatic Mutation and TIC Scores

An increasing number of results indicated that high mutation load closely associates with increased neoantigen expression and gives more chances for the immune system to recognize the tumor. Therefore, we used the “maftools” method to investigate the possible relationships between mutation load and TIC score. To begin, participants in the TCGA-UVM cohort were divided into two groups based on their TIC scores: low and high. The oncoPrint plots summarized the top 20 mutated genes in the low- and high-score groups (**Figure 4E**). GNAQ, GNA11, BAP1, SF3B1, and EIF1AX had 52%, 39%, 19%, 19%, and 19% mutation, respectively, in the high-score samples, while GNA11, BAP1, GNAQ, SF3B1, and DEPDC5 had 54%, 46%, 46%, 31%, and 8%, mutation, respectively, in the low-score samples. Despite tumor burden mutation (TMB) being an indicator for responsiveness to immunotherapy in various tumors, Spearman’s test revealed that TIC scores were not correlated with TMBs (**Figure 4F**). Furthermore, a forest plot indicated that BAP1 highly mutated in the low-score group and EIF1AX highly mutated in the high-score group (**Figure 4G**).

### TIC Scores and Different Phenotypes

**Figure 5:**
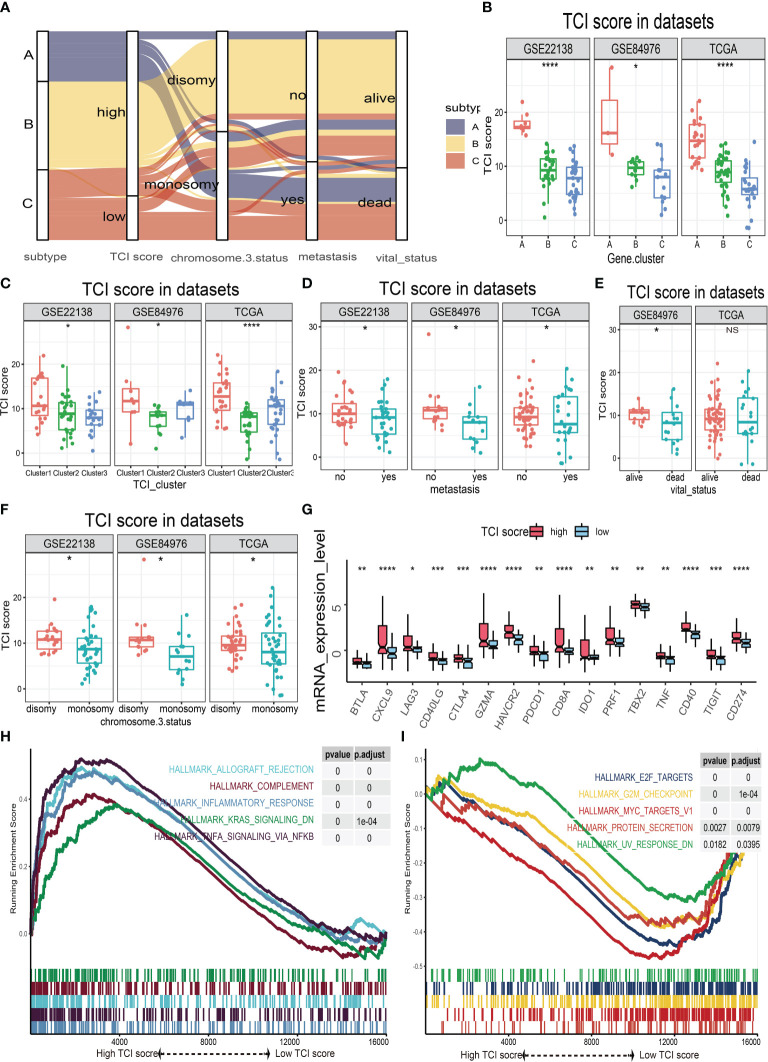
The relationships of TIC scores with clinical parameters, molecular indicators, and biological signal pathways. **(A)** Alluvial plot of immune gene-related clusters with different TIC scores, chromosome 3 status, metastasis, and vital status. **(B)** The TIC score distribution of immune gene-related clusters. **(C)** The TIC score distribution of infiltrated immune cell subtypes. **(D)** The TIC score distribution of metastasis. **(E)** The TIC score distribution of vital status. **(F)** The TIC score distribution of chromosome 3 status. **(G)** Box plots of immune checkpoint-related genes (BTLA, CXCL9, GZMA, CD8A, TBX2, PRF1, TNF, and TIGIT) and immunological activity-related genes (BTLA, CXCL9, GZMA, CD8A, TBX2, PRF1, TNF, and TIGIT) between high-TIC score and low-TIC score groups. **(H)** GSEA plots of significant cancer hallmark pathways enriched in high-TIC score phenotype. **(I)** GSEA plots of significant cancer hallmark pathways enriched in low-TIC score phenotype. *p < 0.05; **p < 0.01; ***p < 0.001; and ****p < 0.0001.

The possible associations of TIC scores with clinical parameters, molecular indicators, and biological signal pathways were further investigated. We firstly classified UM samples into low- and high-score groups, and then Alluvial diagram of TIC scores subgroup illustrated that high TIC score patients who had a large proportion of disomy in chromosome 3 status with no metastasis and alive status (**Figure 5A**). The different distributions of TIC scores in clinical subtypes were also estimated by Kruskal−Wallis or Wilcoxon test, which indicated that immune gene cluster (**Figure 5B**), infiltrated immune cell cluster (**Figure 5C**), metastatic status (**Figure 5D**), vital status (**Figure 5E**), and chromosome 3 status (**Figure 5F**) were intimately associated with TIC scores. Besides, to figure out the immunological activity and tolerance levels of each group, we selected PDCD1, CD274, CTLA4, LAG3, IDO1, HAVCR2, CD40, and CD40LG as immune checkpoint-related genes and picked BTLA, CXCL9, GZMA, CD8A, TBX2, PRF1, TNF, and TIGIT as immunological activity-related genes. Wilcoxon test suggested that the immune checkpoint-related and immunological activity-related genes were all significantly increased in the high-score group (**Figure 5G**). Finally, GSEA was used to discover the various signal pathways that were enriched in each TIC score group. The top five pathways were respectively depicted in the high-score group (**Figure 5H**) and the low-score group (**Figure 5I**) based on the selection standard and the ranking pathways enriched in each phenotype.

### Potential Indicator for Immune Checkpoint Therapy

**Figure 6:**
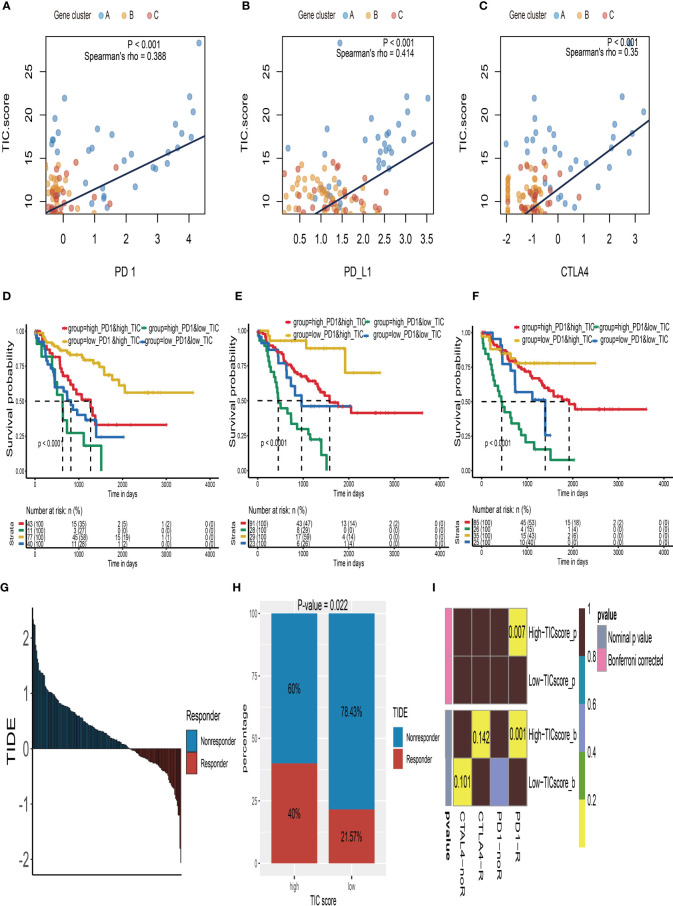
Immune checkpoint therapy response of UM. **(A)** The correlation analysis between TIC scores and PD-1 expressions. **(B)** The correlation analysis between TIC scores and PD-L1 expressions. **(C)** The correlation analysis between TIC scores and CTLA-4 expressions. **(D)** Kaplan–Meier (KM) curve of four groups stratified by the TIC scores and PD-1 expressions. **(E)** KM curve of four groups stratified by the TIC scores and PD-L1 expressions. **(F)** KM curve of four groups stratified by the TIC scores and CTLA-4 expressions. **(G)** The distribution of Tumor Immune Dysfunction and Exclusion (TIDE) scores in all UM patients. **(H)** The different response rate between high-TIC score and low-TIC score groups in terms of immunotherapy. **(I)** Anti-CTLA-4 and anti-PD-1 treatment responses in high-TIC score and low-TIC score groups.

The relationship between TIC scores and the expression patterns of current immune checkpoint inhibitor-associated genes (PD-1, PD-L1, and CTLA-4) was examined further to better understand the possible response to immunotherapy. According to Spearman’s correlation tests, the TIC scores were significantly positively linked to the expression levels of PD-1 (**Figure 6A**), PD-L1 (**Figure 6B**), and CTLA-4 (**Figure 6C**). To investigate the effects of interaction between TIC scores and immune checkpoint genes on patients’ survival, these UM patients were categorized into four subtypes based on TIC scores and immune checkpoint genes, and KM curve analyses were conducted to determine the different survival times among the four subtypes. The log-rank tests indicated that TIC scores can effectively separate patients’ prognosis with contradictory expression levels of PD-1 (**Figure 6D**), PD-L1 (**Figure 6E**), and CTLA-4 (**Figure 6F**). UM patients with the worst prognosis had a low TIC score and a high level of immune checkpoint genes, whereas UM patients with high TIC scores and low levels of immune checkpoint genes had the highest survival rates among the four subtypes.

Because of the intimate connections between TIC scores and immune checkpoint inhibitor-associated genes, we hypothesized that it may be used to predict UM immunotherapy response. Therefore, we firstly used the TIDE module in an online website (http://tide.dfci.harvard.edu/) to estimate TIDE scores for all UM patients (**Figure 6G**). The chi-square test demonstrated that the high TIC score group has a higher response rate (40% vs. 21.57%) than the low-score group (**Figure 6H**). Furthermore, we used subclass mapping analysis to contrast the expression profiles of the high-/low-score subgroups with a previously published dataset of 47 melanoma patients who accepted the immune checkpoint inhibitor therapy (CTLA-4 and PD-1). Surprisingly, we discovered that the high-TIC score group has a better chance of responding to anti-PD-1 therapy. UM patients in the low-TIC score category, on the other hand, are unresponsive to anti-CTLA-4 or anti-PD-1 treatment (**Figure 6I**).

**Figure 7:**
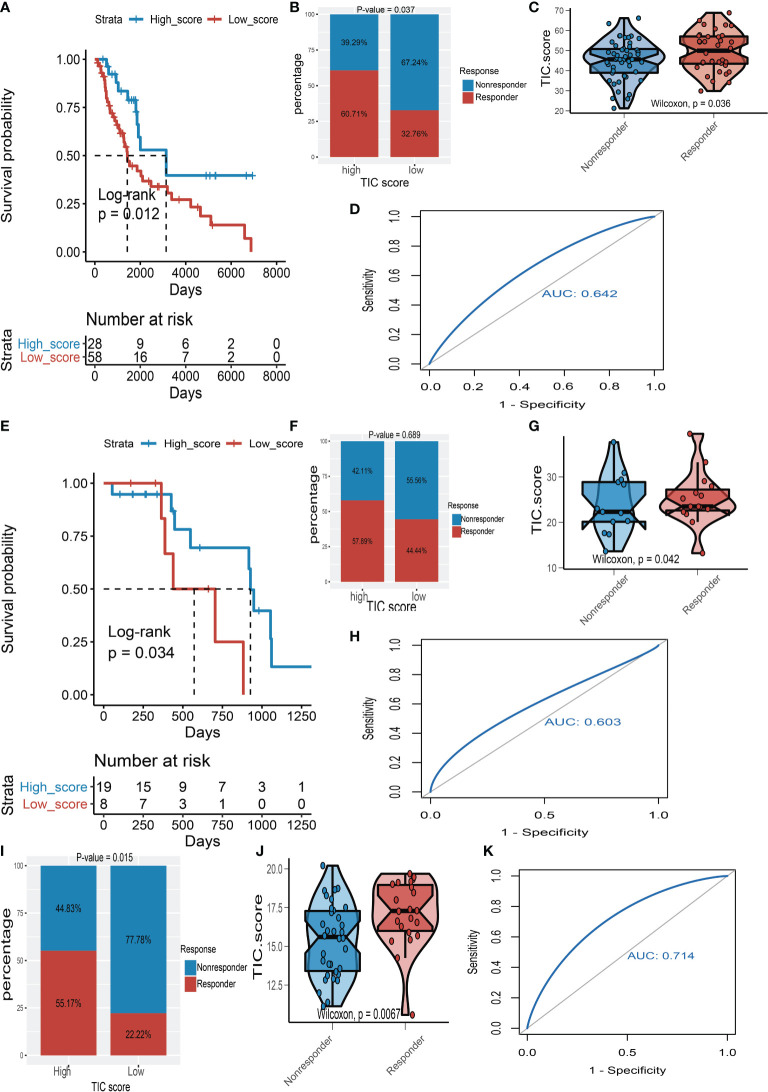
The validation of TIC score for predicting immunotherapy. **(A)** Kaplan–Meier (KM) curve of high-TIC score and low-TIC score groups in TCGA-SKCM. **(B)** Immunotherapy response rate of high- and low-TIC score groups in TCGA-SKCM. **(C)** Distribution of TIC scores between high- and low-TIC score groups in TCGA-SKCM. **(D)** The receiver operating characteristic (ROC) curve of TIC score in TCGA-SKCM. **(E)** KM curve of high-TIC score and low-TIC score groups in GSE78220. **(F)** Immunotherapy response rate of high- and low-TIC score groups in GSE78220. **(G)** Distribution of TIC scores between high- and low-TIC score groups in GSE78220. **(H)** ROC curve of TIC score in GSE78220. **(I)** Immunotherapy response rate of high- and low-TIC score groups in GSE35640. **(J)** Distribution of TIC scores between high- and low-TIC score groups in GSE35640. **(K)** ROC curve of TIC score in GSE35640.

To support our hypothesis, melanoma patients from the TCGA-SKCM, GSE35640, and GSE78220 datasets who received immunotherapy were divided into low- and high-TIC score groups accordingly. In particular, melanoma patients in the high-score category had a longer survival time than those in the low-score group whether in TCGA-SKCM cohort (**Figure 7A**) or the GSE78220 cohort (**Figure 7E**). Besides, the high-score groups had a larger proportion of response rate than low-score groups whether in the TCGA-SKCM cohort (**Figure 7B**), the GSE78220 cohort (**Figure 7F**), and the GSE35640 cohort (**Figure 7I**). The box plots of TIC score distribution in TCGA-SKCM (**Figure 7C**), GSE78220 (**Figure 7G**), and GSE35640 (**Figure 7J**) prove that immunotherapy response groups had higher TIC scores than nonresponse groups. Eventually, ROC curves of TIC scores in TCGA-SKCM (**Figure 7D**), GSE78220 (**Figure 7H**), and GSE35640 (**Figure 7K**) suggested that the TIC scores had a relatively high accuracy to predict the response of immunotherapy.

### Potential Therapeutic Agents for Low-TIC Score Group

**Figure 8:**
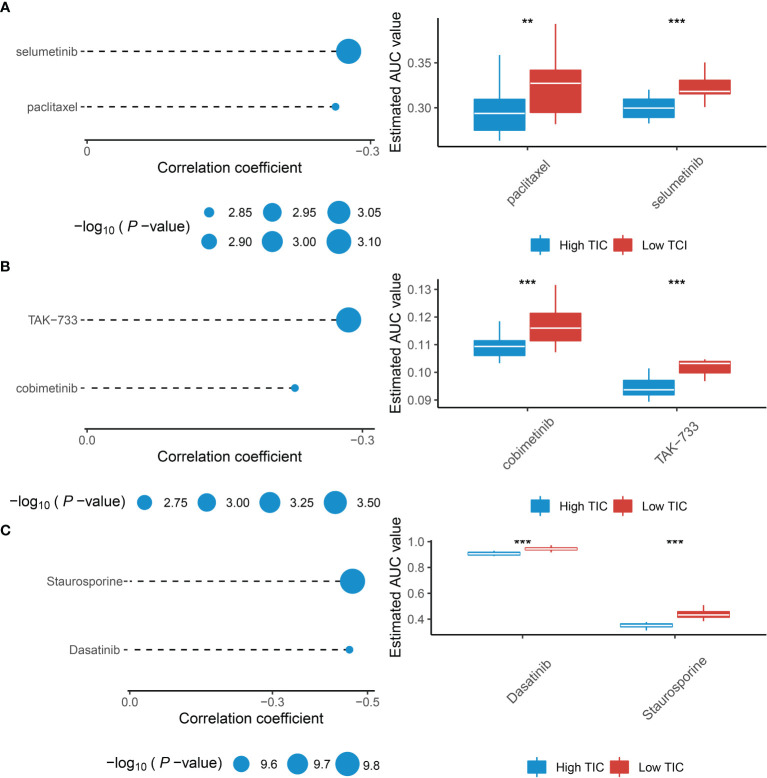
Identification of therapeutic agents for the low-TIC score group. **(A)** The correlation analysis between TIC scores and AUC values of selumetinib and paclitaxel in the CTRP database. **(B)** The correlation analysis between TIC scores and AUC values of cobimetinib and TAK-733 in the PRISM database. **(C)** The correlation analysis between TIC scores and AUC values of dasatinib and staurosporine in the GDSC database. **p < 0.01; ***p < 0.001.

Recently, numerous studies are searching for new promising therapeutic agents for advanced UMs due to resistance to standard chemotherapeutics. Therefore, we used multiple drug response databases to identify possible chemotherapy drugs for low-TIC score patients with poor prognosis. Eventually, we discovered that 6 compounds were negatively associated with TIC scores, which contained selumetinib and paclitaxel in the CTRP database (**Figure 8A**), cobimetinib and TAK-733 in the PRISM database (**Figure 8B**), and dasatinib and staurosporine in the GDSC database (**Figure 8C**). In addition, Wilcoxon test suggested that all of the six compounds had higher AUC values in the low-TIC score group than in the high-TIC score group.

## Discussion

Immune checkpoint blockade (ICB) therapies such as PD-1, PD-L1, and CTLA-4 inhibitors, which are now recognized as a potential treatment strategy, have proven to be extremely effective to prolong the survival time of various advanced tumors like bladder cancer, lung cancer, breast cancer, and cutaneous melanoma (14, 24–26). However, in UM patients, the clinical benefits of ICB therapies are limited (7). Despite the fact that the reasons for the low immunotherapy response in UM patients are unknown, several speculations have been proposed: (1) UMs have a lower TMB than many solid tumors, which means scarce neo-antigens are presented in the surface of tumor tissue and result in failure of recognition for T cells to eradicate cancer cells; (2) the eye is an immune-privileged organ that leads to low immune-mediated inflammation in UM; and (3) limited lymphatic circulation increases tumor antigen retention and final consumption of effective T cells for continued exposure (27–30). Besides, the distinct responses to ICB therapies emphasize the importance of discovering potential predictive signatures. For instance, recent numerous cancer studies have observed a close link between higher TMB and ICI response, indicating that TMB might be a useful signature for predicting ICI response (31–33). However, the fact that TMB does not always associate with ICI responsiveness is a major problem for the usefulness of TMB (34, 35). For example, prostate cancer and uveal melanoma have a low rate to predict ICI response by applying TMB, probably due to relatively low TMB compared to other cancers (36, 37). Therefore, we comprehensively examined the infiltrated immune cells in the UM microenvironment and established a TIC score to stably and precisely predict the prognosis and ICB response of UM patients.

The TME contexture varies greatly among different types of tumors, notably in infiltrated immune cells. Numerous tumor-related studies have demonstrated that TME heterogeneity influences cancer growth and metastasis (38–40). Compared to other solid cancers, the TME contexture in UM is an immunosuppressive status. Lymphocytic infiltrations like increasing infiltration of CD4+ and CD8+ T cells imply a poor prognosis and are frequently linked to metastasis (16, 27). Thus, we distinguished three UM tumor subtypes (Clusters 1 to 3) based on 22 kinds of immune cells. We discovered that UM patients from Cluster 1 were associated with poor survival outcomes and had a high infiltration of CD8+, activated CD4+, follicular helper and gamma delta T cells, M1 macrophages, and resting dendritic cells. These observations were in accordance with previous studies (28, 41). The depiction of clinical characterization illustrated that vital status, metastasis, chromosome 3 status, stage, and histological type were also distinct differences among subtypes. Besides, Cluster 1 had higher expression of PD-1, PD-L1, and CTLA-4 than Cluster 2 and Cluster 3, which indicated that the immune cell-related phenotypes have already determined the response to ICB therapies. Therefore, the gene expression patterns for characterization of immune cell-related phenotypes would be a breakthrough methodology for developing patient-specific individualized treatment. We subsequently explore the DEGs among these subtypes to uncover that these immune-related genes were positively correlated with metabolic pathways such as regulation of lipid metabolic process and regulation of steroid metabolic process, but negatively associated with T-cell activation like lymphocyte proliferation, differentiation, and regulation of T-cell activation. Recent research revealed that the dysregulation of metabolic processes in infiltrated immune cells and tumor cells can limit immune responses to cancer treatment and increase poor prognosis (42, 43). Moreover, the fight for nutrition between tumor cells and infiltrated lymphocytes will lead to immunosuppression (44, 45). Although these pathways suggest an immunosuppressive TME under UM, we observed that Cluster A had a high infiltration of CD8+ T cells, follicular helper T cells, and gamma delta T cells, as well as an increase in PD-1, PD-L1, and CTLA-4 expression which may indicate an ICB-response phenotype.

Then, using various independent datasets, we established and verified a stable TIC score to predict UM prognosis. Following that, we classified UM samples into low- and high-score groups with different survival outcomes, clinical features, and somatic mutations. Despite the fact that there was no significant link between TMB and TIC score, we discovered that the low mutation frequency of BAP1 and the high mutation frequency of EIF1AX exist in the high-score group. It generally suggested that certain gene mutations may cause a specific type of immunological response. For example, lack of BAP1 increased production of chemokines to attract T-cell aggregation, resulting in greater T-cell infiltration in UM (46). When BAP1 is mutated, it causes a significant risk of metastatic disease in UM patients (47). Recently, Figueiredo et al. proved that the absence of BAP1 expression was linked to an immunosuppressive TME in UM (48). Besides, mutations in EIF1AX were found to play a protective role in UM metastasis (49). Martin et al. reported that UM with mutations in EIF1AX had a better prognosis than tumors without mutations in these genes (50). Taking all into consideration, these lines of evidence suggest that our TIC score is credible and in line with previous studies. Subsequently, we performed GSEA to show that the high-TIC score subtype contains various immunological responses such as allograft rejection, complement, inflammatory response, and TNFA signal *via* NFKB. Furthermore, the high-TIC score group has a greater expression of immune checkpoint-related molecules. As a result, it is simple to see why high-TIC score UM patients have a greater survival rate than low-score ones.

Surprisingly, we discovered a substantially positive connection between the TIC score and the expression of PD-1, PD-L1, and CTLA-4. The crosstalk of TIC score and immune checkpoint genes (PD-1, PD-L1, and CTLA-4) for survival analysis revealed a mutually supportive effect on patients’ prognosis. Therefore, determining the utility of TIC score for predicting ICB responses is critical. We observed that high-TIC score UM patients with low TIDE scores are more likely to respond to ICB therapy. *Via* subclass mapping analysis, we interestingly noticed that the high-TIC score group is more likely to respond to anti-PD1 therapy. To support our observation, patients who underwent immunotherapy in the TCGA-SKCM, GSE35640, and GSE78220 cohorts were analyzed accordingly. The patients who responded to immunotherapy had higher TIC scores than those with no response. KM curves also proved the prognostic utility of TIC score. Consequently, we speculated that the TIC scores can directly reflect the complex TME contexture and UM patients with high TIC scores may benefit from ICB therapies.

Actually, only a small number of UM patients respond to immunotherapies in clinical trials. Therefore, an investigation of potential compounds for UM treatment is necessary. *Via* correlation analysis, we identified that selumetinib, paclitaxel, cobimetinib, TAK-733, dasatinib, and staurosporine were more effective in treating low-TIC score UM patients. Paclitaxel is a common chemotherapy drug for solid tumors (51). It is currently used as a second-line chemotherapeutic medication in the treatment of metastatic melanoma (52). Selumetinib and TAK-733 are elective allosteric inhibitors for MEK1 and MEK2 (53, 54). Komatsubara et al. reported that selumetinib can significantly improve the progression-free survival of metastatic UM in contrast to the traditional chemotherapy group (55). Cobimetinib is also a MEK inhibitor, which had been approved by the FDA and used as a first-line treatment for unresectable advanced melanoma (56). Besides, dasatinib targets SRC kinase to inhibit the proliferation and invasion of melanoma cell lines *in vitro* (57). As a result, it is acceptable to suppose that these compounds might be used as adjunctive therapies or in combination with other treatments for UM.

## Conclusion

In conclusion, we extensively examined the landscape of infiltrated immune cell characterization in UM and provided a comprehensive view of immune response regulation. We also established a robust TIC score signature that closely associated with UM heterogeneity and ICB response complexity. The high-TIC score patients have a better prognosis and may have more immune therapeutic advantages. Thus, our study has a crucial implication for the systematic evaluation of UM immune patterns.

## Data Availability Statement

The datasets presented in this study can be found in online repositories. The names of the repository/repositories and accession number(s) can be found in the article/**Supplementary Material**.

## Author Contributions

XL was in charge of writing. MD was in duty of writing and editing. YL was in charge of project administration and funding acquisition. All authors contributed to the article and approved the submitted version.

## Conflict of Interest

The authors declare that the research was conducted in the absence of any commercial or financial relationships that could be construed as a potential conflict of interest.

## Publisher’s Note

All claims expressed in this article are solely those of the authors and do not necessarily represent those of their affiliated organizations, or those of the publisher, the editors and the reviewers. Any product that may be evaluated in this article, or claim that may be made by its manufacturer, is not guaranteed or endorsed by the publisher.
